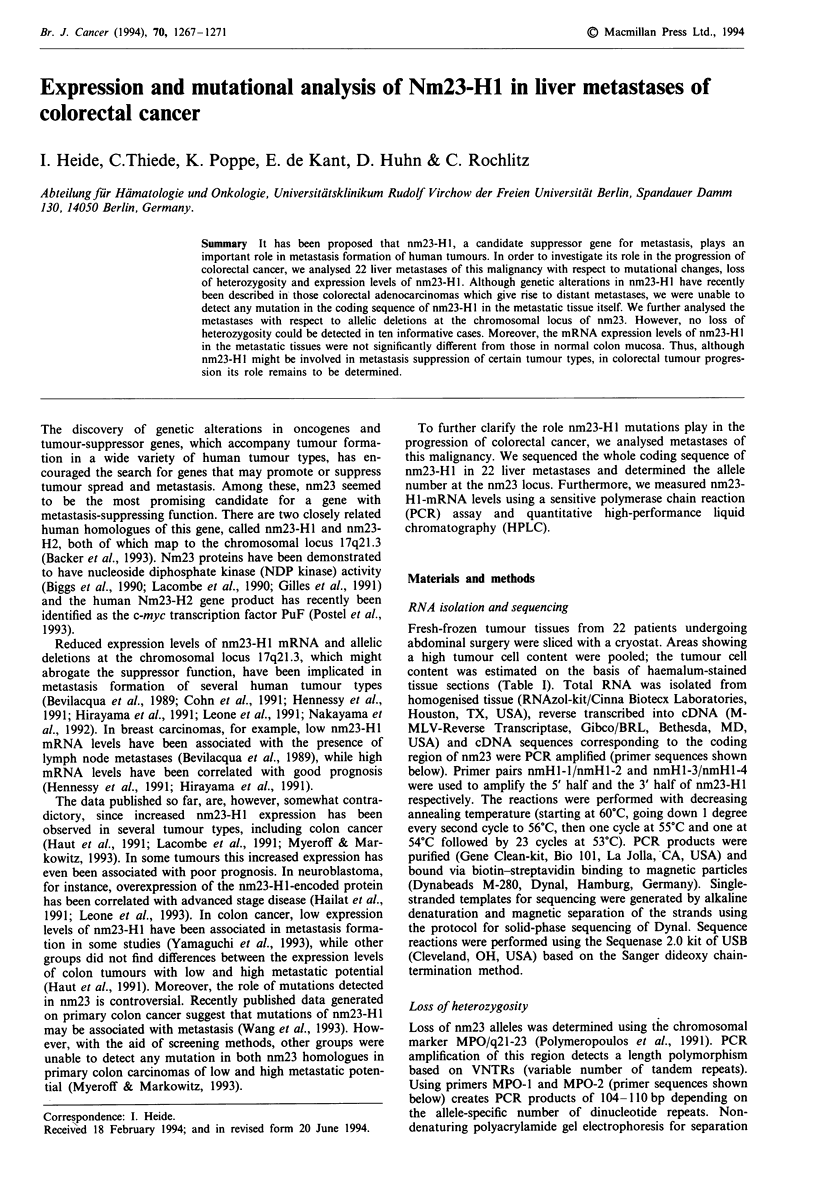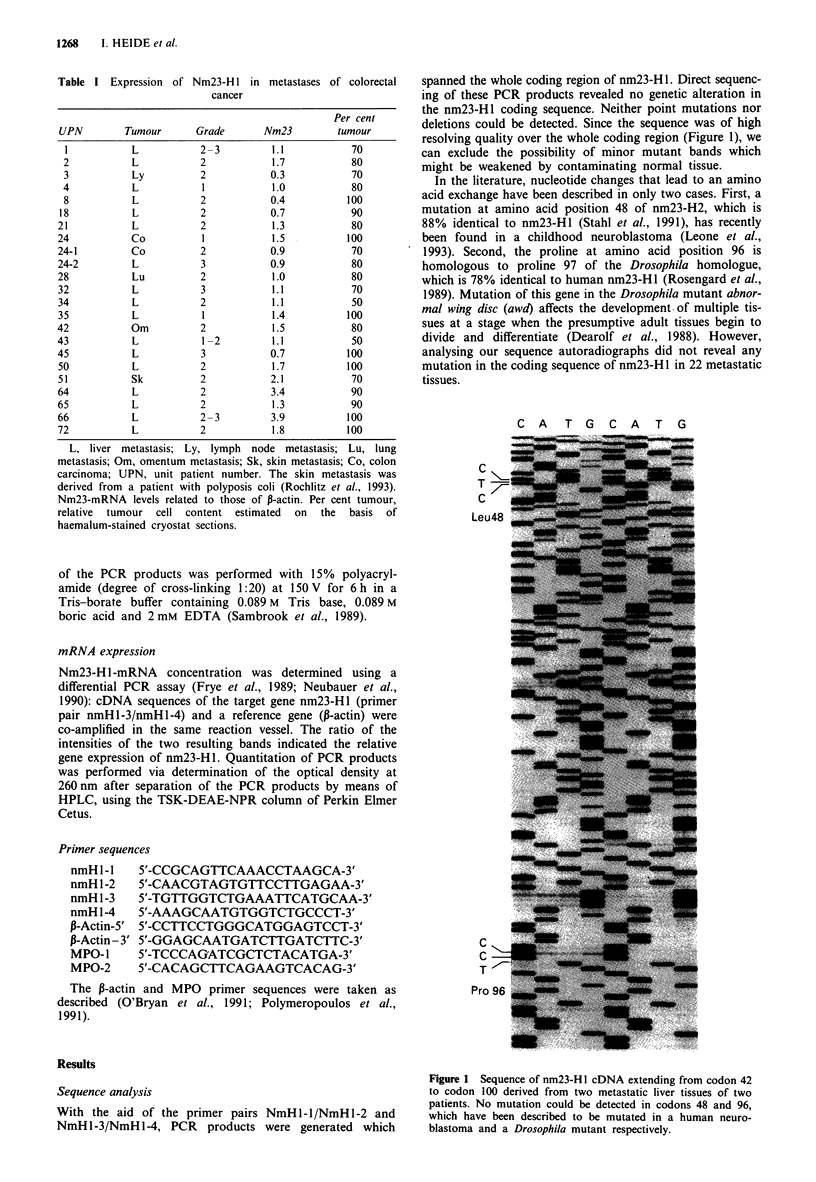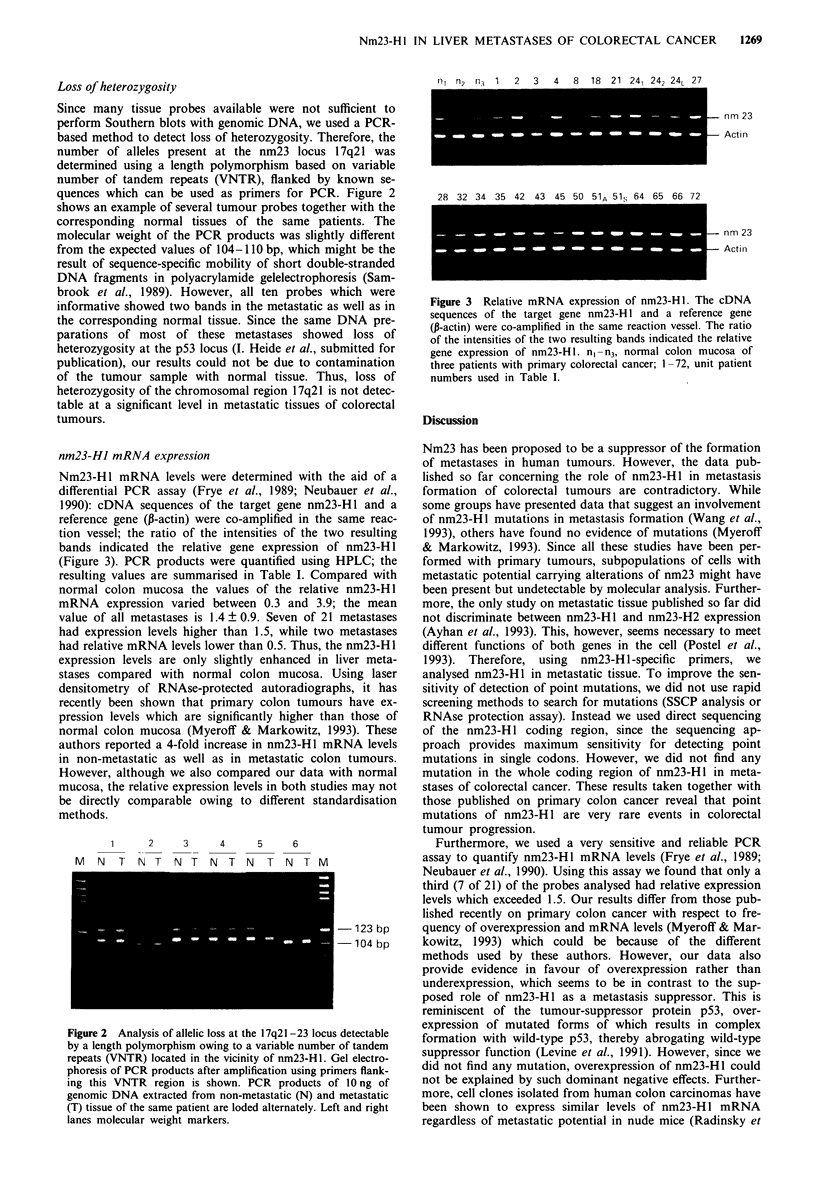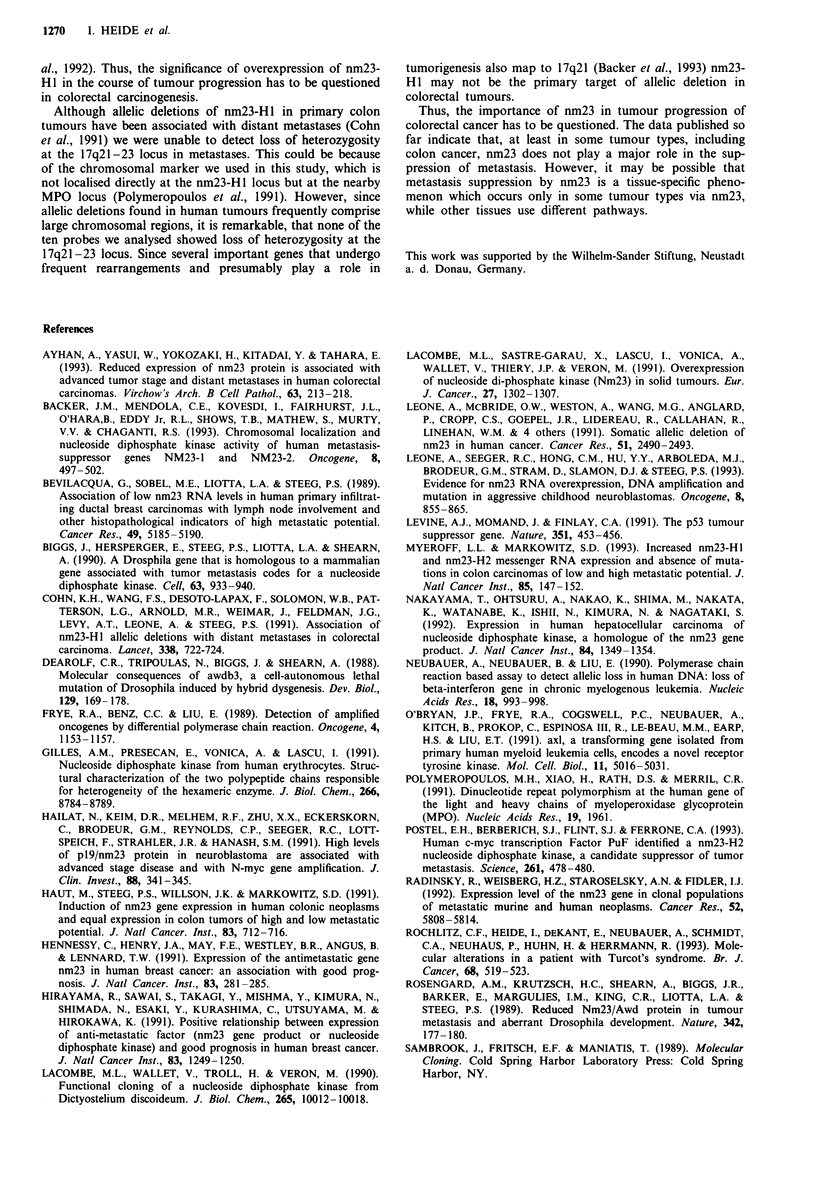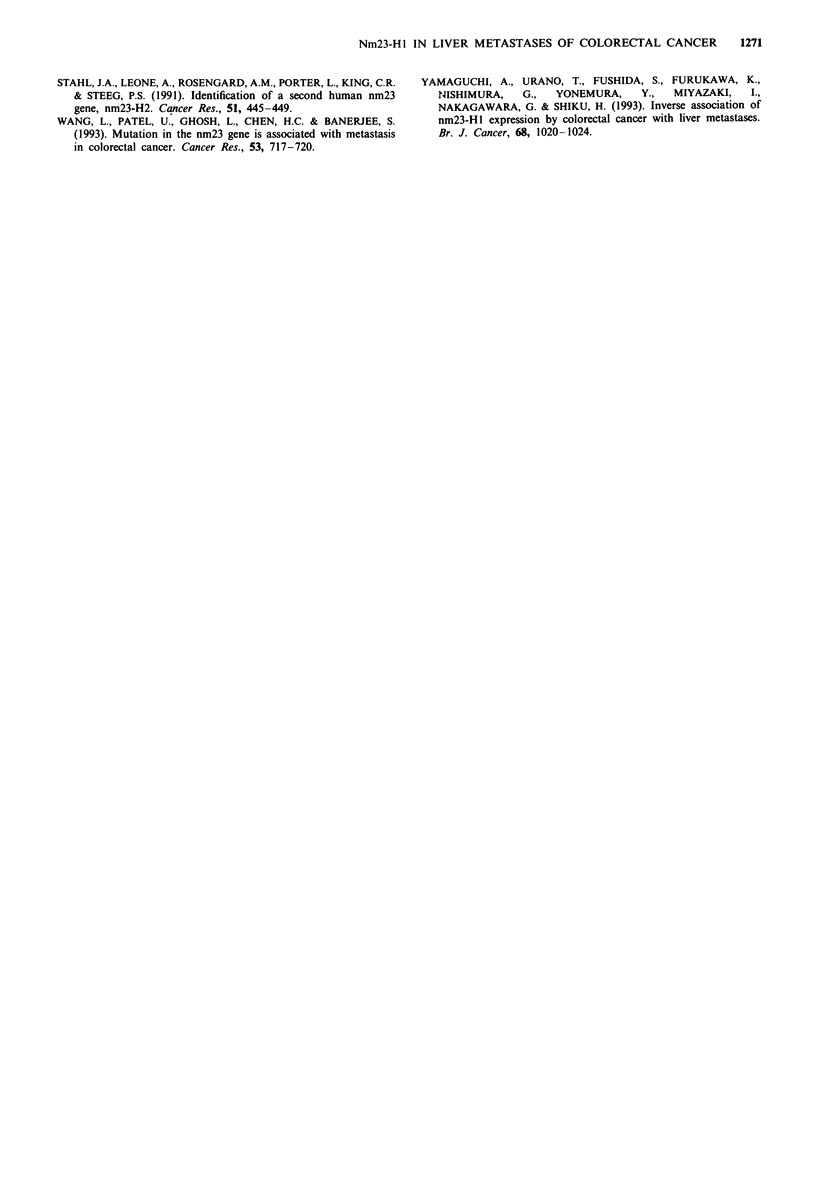# Expression and mutational analysis of Nm23-H1 in liver metastases of colorectal cancer.

**DOI:** 10.1038/bjc.1994.485

**Published:** 1994-12

**Authors:** I. Heide, C. Thiede, K. Poppe, E. de Kant, D. Huhn, C. Rochlitz

**Affiliations:** Abteilung für Hämatologie und Onkologie, Universitätsklinikum Rudolf Virchow, Freien Universität Berlin, Germany.

## Abstract

**Images:**


Br. J. Cancer (1994), 70, 1267–1271 © Macmillan Press Ltd., 1994

### Expression and mutational analysis of Nm23-H1 in liver metastases of colorectal cancer

I. Heide, C.Thiede, K. Poppe, E. de Kant, D. Huhn & C. Rochlitz

*Abteilung für Hämatologie und Onkologie, Universitätsklinikum Rudolf Virchow der Freien Universität Berlin, Spandauer Damm 130, 14050 Berlin, Germany.*

**Summary** It has been proposed that nm23-H1, a candidate suppressor gene for metastasis, plays an important role in metastasis formation of human tumours. In order to investigate its role in the progression of colorectal cancer, we analysed 22 liver metastases of this malignancy with respect to mutational changes, loss of heterozygosity and expression levels of nm23-H1. Although genetic alterations in nm23-H1 have recently been described in those colorectal adenocarcinomas which give rise to distant metastases, we were unable to detect any mutation in the coding sequence of nm23-H1 in the metastatic tissue itself. We further analysed the metastases with respect to allelic deletions at the chromosomal locus of nm23. However, no loss of heterozygosity could be detected in ten informative cases. Moreover, the mRNA expression levels of nm23-H1 in the metastatic tissues were not significantly different from those in normal colon mucosa. Thus, although nm23-H1 might be involved in metastasis suppression of certain tumour types, in colorectal tumour progression its role remains to be determined.

The discovery of genetic alterations in oncogenes and tumour-suppressor genes, which accompany tumour formation in a wide variety of human tumour types, has encouraged the search for genes that may promote or suppress tumour spread and metastasis. Among these, nm23 seemed to be the most promising candidate for a gene with metastasis-suppressing function. There are two closely related human homologues of this gene, called nm23-H1 and nm23-H2, both of which map to the chromosomal locus 17q21.3 (Backer *et al.*, 1993). Nm23 proteins have been demonstrated to have nucleoside diphosphate kinase (NDP kinase) activity (Biggs *et al.*, 1990; Lacombe *et al.*, 1990; Gilles *et al.*, 1991) and the human Nm23-H2 gene product has recently been identified as the c-*myc* transcription factor PuF (Postel *et al.*, 1993).

Reduced expression levels of nm23-H1 mRNA and allelic deletions at the chromosomal locus 17q21.3, which might abrogate the suppressor function, have been implicated in metastasis formation of several human tumour types (Bevilacqua *et al.*, 1989; Cohn *et al.*, 1991; Hennessy *et al.*, 1991; Hirayama *et al.*, 1991; Leone *et al.*, 1991; Nakayama *et al.*, 1992). In breast carcinomas, for example, low nm23-H1 mRNA levels have been associated with the presence of lymph node metastases (Bevilacqua *et al.*, 1989), while high mRNA levels have been correlated with good prognosis (Hennessy *et al.*, 1991; Hirayama *et al.*, 1991).

The data published so far, are, however, somewhat contradictory, since increased nm23-H1 expression has been observed in several tumour types, including colon cancer (Haut *et al.*, 1991; Lacombe *et al.*, 1991; Myeroff & Markowitz, 1993). In some tumours this increased expression has even been associated with poor prognosis. In neuroblastoma, for instance, overexpression of the nm23-H1-encoded protein has been correlated with advanced stage disease (Hailat *et al.*, 1991; Leone *et al.*, 1993). In colon cancer, low expression levels of nm23-H1 have been associated in metastasis formation in some studies (Yamaguchi *et al.*, 1993), while other groups did not find differences between the expression levels of colon tumours with low and high metastatic potential (Haut *et al.*, 1991). Moreover, the role of mutations detected in nm23 is controversial. Recently published data generated on primary colon cancer suggest that mutations of nm23-H1 may be associated with metastasis (Wang *et al.*, 1993). However, with the aid of screening methods, other groups were unable to detect any mutation in both nm23 homologues in primary colon carcinomas of low and high metastatic potential (Myeroff & Markowitz, 1993).

Correspondence: I. Heide.
Received 18 February 1994; and in revised form 20 June 1994.

To further clarify the role nm23-H1 mutations play in the progression of colorectal cancer, we analysed metastases of this malignancy. We sequenced the whole coding sequence of nm23-H1 in 22 liver metastases and determined the allele number at the nm23 locus. Furthermore, we measured nm23-H1-mRNA levels using a sensitive polymerase chain reaction (PCR) assay and quantitative high-performance liquid chromatography (HPLC).

### Materials and methods

### *RNA isolation and sequencing*

Fresh-frozen tumour tissues from 22 patients undergoing abdominal surgery were sliced with a cryostat. Areas showing a high tumour cell content were pooled; the tumour cell content was estimated on the basis of haemalum-stained tissue sections (Table I). Total RNA was isolated from homogenised tissue (RNAzol-kit/Cinna Biotecx Laboratories, Houston, TX, USA), reverse transcribed into cDNA (M-MLV-Reverse Transcriptase, Gibco/BRL, Bethesda, MD, USA) and cDNA sequences corresponding to the coding region of nm23 were PCR amplified (primer sequences shown below). Primer pairs nmH1-1/nmH1-2 and nmH1-3/nmH1-4 were used to amplify the 5′ half and the 3′ half of nm23-H1 respectively. The reactions were performed with decreasing annealing temperature (starting at 60°C, going down 1 degree every second cycle to 56°C, then one cycle at 55°C and one at 54°C followed by 23 cycles at 53°C). PCR products were purified (Gene Clean-kit, Bio 101, La Jolla, CA, USA) and bound via biotin–streptavidin binding to magnetic particles (Dynabeads M-280, Dynal, Hamburg, Germany). Single-stranded templates for sequencing were generated by alkaline denaturation and magnetic separation of the strands using the protocol for solid-phase sequencing of Dynal. Sequence reactions were performed using the Sequenase 2.0 kit of USB (Cleveland, OH, USA) based on the Sanger dideoxy chain-termination method.

### *Loss of heterozygosity*

Loss of nm23 alleles was determined using the chromosomal marker MPO/q21-23 (Polymeropoulos *et al.*, 1991). PCR amplification of this region detects a length polymorphism based on VNTRs (variable number of tandem repeats). Using primers MPO-1 and MPO-2 (primer sequences shown below) creates PCR products of 104–110 bp depending on the allele-specific number of dinucleotide repeats. Non-denaturing polyacrylamide gel electrophoresis for separation

**Table I** Expression of Nm23-H1 in metastases of colorectal cancer

| *UPN* | *Tumour* | *Grade* | *Nm23* | *Per cent tumour* |
|---|---|---|---|---|
| 1 | L | 2–3 | 1.1 | 70 |
| 2 | L | 2 | 1.7 | 80 |
| 3 | Ly | 2 | 0.3 | 70 |
| 4 | L | 1 | 1.0 | 80 |
| 8 | L | 2 | 0.4 | 100 |
| 18 | L | 2 | 0.7 | 90 |
| 21 | L | 2 | 1.3 | 80 |
| 24 | Co | 1 | 1.5 | 100 |
| 24-1 | Co | 2 | 0.9 | 70 |
| 24-2 | L | 3 | 0.9 | 80 |
| 28 | Lu | 2 | 1.0 | 80 |
| 32 | L | 3 | 1.1 | 70 |
| 34 | L | 2 | 1.1 | 50 |
| 35 | L | 1 | 1.4 | 100 |
| 42 | Om | 2 | 1.5 | 80 |
| 43 | L | 1–2 | 1.1 | 50 |
| 45 | L | 3 | 0.7 | 100 |
| 50 | L | 2 | 1.7 | 100 |
| 51 | Sk | 2 | 2.1 | 70 |
| 64 | L | 2 | 3.4 | 90 |
| 65 | L | 2 | 1.3 | 90 |
| 66 | L | 2–3 | 3.9 | 100 |
| 72 | L | 2 | 1.8 | 100 |

L, liver metastasis; Ly, lymph node metastasis; Lu, lung metastasis; Om, omentum metastasis; Sk, skin metastasis; Co, colon carcinoma; UPN, unit patient number. The skin metastasis was derived from a patient with polyposis coli (Rochlitz et al., 1993). Nm23-mRNA levels related to those of β-actin. Per cent tumour, relative tumour cell content estimated on the basis of haemalum-stained cryostat sections.

of the PCR products was performed with 15% polyacrylamide (degree of cross-linking 1:20) at 150 V for 6 h in a Tris–borate buffer containing 0.089 M Tris base, 0.089 M boric acid and 2 mM EDTA (Sambrook et al., 1989).

### *mRNA expression*

Nm23-H1-mRNA concentration was determined using a differential PCR assay (Frye et al., 1989; Neubauer et al., 1990): cDNA sequences of the target gene nm23-H1 (primer pair nmH1-3/nmH1-4) and a reference gene (β-actin) were co-amplified in the same reaction vessel. The ratio of the intensities of the two resulting bands indicated the relative gene expression of nm23-H1. Quantitation of PCR products was performed via determination of the optical density at 260 nm after separation of the PCR products by means of HPLC, using the TSK-DEAE-NPR column of Perkin Elmer Cetus.

### *Primer sequences*

| | |
|---|---|
| nmH1-1 | 5′-CCGCAGTTCAAACCTAAGCA-3′ |
| nmH1-2 | 5′-CAACGTAGTGTTCCTTGAGAA-3′ |
| nmH1-3 | 5′-TGTTGGTCTGAAATTCATGCAA-3′ |
| nmH1-4 | 5′-AAAGCAATGTGGTCTGCCCT-3′ |
| β-Actin-5′ | 5′-CCTTCCTGGGCATGGAGTCCT-3′ |
| β-Actin-3′ | 5′-GGAGCAATGATCTTGATCTTC-3′ |
| MPO-1 | 5′-TCCCAGATCGCTCTACATGA-3′ |
| MPO-2 | 5′-CACAGCTTCAGAAGTCACAG-3′ |

The β-actin and MPO primer sequences were taken as described (O'Bryan et al., 1991; Polymeropoulos et al., 1991).

### Results

### *Sequence analysis*

With the aid of the primer pairs NmH1-1/NmH1-2 and NmH1-3/NmH1-4, PCR products were generated which spanned the whole coding region of nm23-H1. Direct sequencing of these PCR products revealed no genetic alteration in the nm23-H1 coding sequence. Neither point mutations nor deletions could be detected. Since the sequence was of high resolving quality over the whole coding region (Figure 1), we can exclude the possibility of minor mutant bands which might be weakened by contaminating normal tissue.

In the literature, nucleotide changes that lead to an amino acid exchange have been described in only two cases. First, a mutation at amino acid position 48 of nm23-H2, which is 88% identical to nm23-H1 (Stahl et al., 1991), has recently been found in a childhood neuroblastoma (Leone et al., 1993). Second, the proline at amino acid position 96 is homologous to proline 97 of the *Drosophila* homologue, which is 78% identical to human nm23-H1 (Rosengard et al., 1989). Mutation of this gene in the *Drosophila* mutant *abnormal wing disc* (*awd*) affects the development of multiple tissues at a stage when the presumptive adult tissues begin to divide and differentiate (Dearolf et al., 1988). However, analysing our sequence autoradiographs did not reveal any mutation in the coding sequence of nm23-H1 in 22 metastatic tissues.

**Figure 1** Sequence of nm23-H1 cDNA extending from codon 42 to codon 100 derived from two metastatic liver tissues of two patients. No mutation could be detected in codons 48 and 96, which have been described to be mutated in a human neuroblastoma and a *Drosophila* mutant respectively.

*Loss of heterozygosity*

Since many tissue probes available were not sufficient to perform Southern blots with genomic DNA, we used a PCR-based method to detect loss of heterozygosity. Therefore, the number of alleles present at the nm23 locus 17q21 was determined using a length polymorphism based on variable number of tandem repeats (VNTR), flanked by known sequences which can be used as primers for PCR. Figure 2 shows an example of several tumour probes together with the corresponding normal tissues of the same patients. The molecular weight of the PCR products was slightly different from the expected values of 104–110 bp, which might be the result of sequence-specific mobility of short double-stranded DNA fragments in polyacrylamide gelelectrophoresis (Sambrook *et al.*, 1989). However, all ten probes which were informative showed two bands in the metastatic as well as in the corresponding normal tissue. Since the same DNA preparations of most of these metastases showed loss of heterozygosity at the p53 locus (I. Heide *et al.*, submitted for publication), our results could not be due to contamination of the tumour sample with normal tissue. Thus, loss of heterozygosity of the chromosomal region 17q21 is not detectable at a significant level in metastatic tissues of colorectal tumours.

*nm23-H1 mRNA expression*

Nm23-H1 mRNA levels were determined with the aid of a differential PCR assay (Frye *et al.*, 1989; Neubauer *et al.*, 1990): cDNA sequences of the target gene nm23-H1 and a reference gene (β-actin) were co-amplified in the same reaction vessel; the ratio of the intensities of the two resulting bands indicated the relative gene expression of nm23-H1 (Figure 3). PCR products were quantified using HPLC; the resulting values are summarised in Table I. Compared with normal colon mucosa the values of the relative nm23-H1 mRNA expression varied between 0.3 and 3.9; the mean value of all metastases is $1.4 \pm 0.9$. Seven of 21 metastases had expression levels higher than 1.5, while two metastases had relative mRNA levels lower than 0.5. Thus, the nm23-H1 expression levels are only slightly enhanced in liver metastases compared with normal colon mucosa. Using laser densitometry of RNAse-protected autoradiographs, it has recently been shown that primary colon tumours have expression levels which are significantly higher than those of normal colon mucosa (Myeroff & Markowitz, 1993). These authors reported a 4-fold increase in nm23-H1 mRNA levels in non-metastatic as well as in metastatic colon tumours. However, although we also compared our data with normal mucosa, the relative expression levels in both studies may not be directly comparable owing to different standardisation methods.

**Figure 2** Analysis of allelic loss at the 17q21–23 locus detectable by a length polymorphism owing to a variable number of tandem repeats (VNTR) located in the vicinity of nm23-H1. Gel electrophoresis of PCR products after amplification using primers flanking this VNTR region is shown. PCR products of 10 ng of genomic DNA extracted from non-metastatic (N) and metastatic (T) tissue of the same patient are loded alternately. Left and right lanes molecular weight markers.

**Figure 3** Relative mRNA expression of nm23-H1. The cDNA sequences of the target gene nm23-H1 and a reference gene (β-actin) were co-amplified in the same reaction vessel. The ratio of the intensities of the two resulting bands indicated the relative gene expression of nm23-H1. $n_1 - n_3$, normal colon mucosa of three patients with primary colorectal cancer; 1–72, unit patient numbers used in Table I.

### Discussion

Nm23 has been proposed to be a suppressor of the formation of metastases in human tumours. However, the data published so far concerning the role of nm23-H1 in metastasis formation of colorectal tumours are contradictory. While some groups have presented data that suggest an involvement of nm23-H1 mutations in metastasis formation (Wang *et al.*, 1993), others have found no evidence of mutations (Myeroff & Markowitz, 1993). Since all these studies have been performed with primary tumours, subpopulations of cells with metastatic potential carrying alterations of nm23 might have been present but undetectable by molecular analysis. Furthermore, the only study on metastatic tissue published so far did not discriminate between nm23-H1 and nm23-H2 expression (Ayhan *et al.*, 1993). This, however, seems necessary to meet different functions of both genes in the cell (Postel *et al.*, 1993). Therefore, using nm23-H1-specific primers, we analysed nm23-H1 in metastatic tissue. To improve the sensitivity of detection of point mutations, we did not use rapid screening methods to search for mutations (SSCP analysis or RNAse protection assay). Instead we used direct sequencing of the nm23-H1 coding region, since the sequencing approach provides maximum sensitivity for detecting point mutations in single codons. However, we did not find any mutation in the whole coding region of nm23-H1 in metastases of colorectal cancer. These results taken together with those published on primary colon cancer reveal that point mutations of nm23-H1 are very rare events in colorectal tumour progression.

Furthermore, we used a very sensitive and reliable PCR assay to quantify nm23-H1 mRNA levels (Frye *et al.*, 1989; Neubauer *et al.*, 1990). Using this assay we found that only a third (7 of 21) of the probes analysed had relative expression levels which exceeded 1.5. Our results differ from those published recently on primary colon cancer with respect to frequency of overexpression and mRNA levels (Myeroff & Markowitz, 1993) which could be because of the different methods used by these authors. However, our data also provide evidence in favour of overexpression rather than underexpression, which seems to be in contrast to the supposed role of nm23-H1 as a metastasis suppressor. This is reminiscent of the tumour-suppressor protein p53, overexpression of mutated forms of which results in complex formation with wild-type p53, thereby abrogating wild-type suppressor function (Levine *et al.*, 1991). However, since we did not find any mutation, overexpression of nm23-H1 could not be explained by such dominant negative effects. Furthermore, cell clones isolated from human colon carcinomas have been shown to express similar levels of nm23-H1 mRNA regardless of metastatic potential in nude mice (Radinsky *et*

al., 1992). Thus, the significance of overexpression of nm23-H1 in the course of tumour progression has to be questioned in colorectal carcinogenesis.

Although allelic deletions of nm23-H1 in primary colon tumours have been associated with distant metastases (Cohn et al., 1991) we were unable to detect loss of heterozygosity at the 17q21-23 locus in metastases. This could be because of the chromosomal marker we used in this study, which is not localised directly at the nm23-H1 locus but at the nearby MPO locus (Polymeropoulos et al., 1991). However, since allelic deletions found in human tumours frequently comprise large chromosomal regions, it is remarkable, that none of the ten probes we analysed showed loss of heterozygosity at the 17q21-23 locus. Since several important genes that undergo frequent rearrangements and presumably play a role in tumorigenesis also map to 17q21 (Backer et al., 1993) nm23-H1 may not be the primary target of allelic deletion in colorectal tumours.

Thus, the importance of nm23 in tumour progression of colorectal cancer has to be questioned. The data published so far indicate that, at least in some tumour types, including colon cancer, nm23 does not play a major role in the suppression of metastasis. However, it may be possible that metastasis suppression by nm23 is a tissue-specific phenomenon which occurs only in some tumour types via nm23, while other tissues use different pathways.

This work was supported by the Wilhelm-Sander Stiftung, Neustadt a. d. Donau, Germany.

### References

AYHAN, A., YASUI, W., YOKOZAKI, H., KITADAI, Y. & TAHARA, E. (1993). Reduced expression of nm23 protein is associated with advanced tumor stage and distant metastases in human colorectal carcinomas. *Virchow's Arch. B Cell Pathol.*, **63**, 213-218.

BACKER, J.M., MENDOLA, C.E., KOVESDI, I., FAIRHURST, J.L., O'HARA,B., EDDY Jr, R.L., SHOWS, T.B., MATHEW, S., MURTY, V.V. & CHAGANTI, R.S. (1993). Chromosomal localization and nucleoside diphosphate kinase activity of human metastasis-suppressor genes NM23-1 and NM23-2. *Oncogene*, **8**, 497-502.

BEVILACQUA, G., SOBEL, M.E., LIOTTA, L.A. & STEEG, P.S. (1989). Association of low nm23 RNA levels in human primary infiltrating ductal breast carcinomas with lymph node involvement and other histopathological indicators of high metastatic potential. *Cancer Res.*, **49**, 5185-5190.

BIGGS, J., HERSPERGER, E., STEEG, P.S., LIOTTA, L.A. & SHEARN, A. (1990). A Drosphila gene that is homologous to a mammalian gene associated with tumor metastasis codes for a nucleoside diphosphate kinase. *Cell*, **63**, 933-940.

COHN, K.H., WANG, F.S., DESOTO-LAPAX, F., SOLOMON, W.B., PATTERSON, L.G., ARNOLD, M.R., WEIMAR, J., FELDMAN, J.G., LEVY, A.T., LEONE, A. & STEEG, P.S. (1991). Association of nm23-H1 allelic deletions with distant metastases in colorectal carcinoma. *Lancet*, **338**, 722-724.

DEAROLF, C.R., TRIPOULAS, N., BIGGS, J. & SHEARN, A. (1988). Molecular consequences of awdb3, a cell-autonomous lethal mutation of Drosophila induced by hybrid dysgenesis. *Dev. Biol.*, **129**, 169-178.

FRYE, R.A., BENZ, C.C. & LIU, E. (1989). Detection of amplified oncogenes by differential polymerase chain reaction. *Oncogene*, **4**, 1153-1157.

GILLES, A.M., PRESECAN, E., VONICA, A. & LASCU, I. (1991). Nucleoside diphosphate kinase from human erythrocytes. Structural characterization of the two polypeptide chains responsible for heterogeneity of the hexameric enzyme. *J. Biol. Chem.*, **266**, 8784-8789.

HAILAT, N., KEIM, D.R., MELHEM, R.F., ZHU, X.X., ECKERSKORN, C., BRODEUR, G.M., REYNOLDS, C.P., SEEGER, R.C., LOTTSPEICH, F., STRAHLER, J.R. & HANASH, S.M. (1991). High levels of p19/nm23 protein in neuroblastoma are associated with advanced stage disease and with N-myc gene amplification. *J. Clin. Invest.*, **88**, 341-345.

HAUT, M., STEEG, P.S., WILLSON, J.K. & MARKOWITZ, S.D. (1991). Induction of nm23 gene expression in human colonic neoplasms and equal expression in colon tumors of high and low metastatic potential. *J. Natl Cancer. Inst.*, **83**, 712-716.

HENNESSY, C., HENRY, J.A., MAY, F.E., WESTLEY, B.R., ANGUS, B. & LENNARD, T.W. (1991). Expression of the antimetastatic gene nm23 in human breast cancer: an association with good prognosis. *J. Natl Cancer. Inst.*, **83**, 281-285.

HIRAYAMA, R., SAWAI, S., TAKAGI, Y., MISHMA, Y., KIMURA, N., SHIMADA, N., ESAKI, Y., KURASHIMA, C., UTSUYAMA, M. & HIROKAWA, K. (1991). Positive relationship between expression of anti-metastatic factor (nm23 gene product or nucleoside diphosphate kinase) and good prognosis in human breast cancer. *J. Natl Cancer Inst.*, **83**, 1249-1250.

LACOMBE, M.L., WALLET, V., TROLL, H. & VERON, M. (1990). Functional cloning of a nucleoside diphosphate kinase from Dictyostelium discoideum. *J. Biol. Chem.*, **265**, 10012-10018.

LACOMBE, M.L., SASTRE-GARAU, X., LASCU, I., VONICA, A., WALLET, V., THIERY, J.P. & VERON, M. (1991). Overexpression of nucleoside di-phosphate kinase (Nm23) in solid tumours. *Eur. J. Cancer.*, **27**, 1302-1307.

LEONE, A., McBRIDE, O.W., WESTON, A., WANG, M.G., ANGLARD, P., CROPP, C.S., GOEPEL, J.R., LIDEREAU, R., CALLAHAN, R., LINEHAN, W.M. & 4 others (1991). Somatic allelic deletion of nm23 in human cancer. *Cancer Res.*, **51**, 2490-2493.

LEONE, A., SEEGER, R.C., HONG, C.M., HU, Y.Y., ARBOLEDA, M.J., BRODEUR, G.M., STRAM, D., SLAMON, D.J. & STEEG, P.S. (1993). Evidence for nm23 RNA overexpression, DNA amplification and mutation in aggressive childhood neuroblastomas. *Oncogene*, **8**, 855-865.

LEVINE, A.J., MOMAND, J. & FINLAY, C.A. (1991). The p53 tumour suppressor gene. *Nature*, **351**, 453-456.

MYEROFF, L.L. & MARKOWITZ, S.D. (1993). Increased nm23-H1 and nm23-H2 messenger RNA expression and absence of mutations in colon carcinomas of low and high metastatic potential. *J. Natl Cancer Inst.*, **85**, 147-152.

NAKAYAMA, T., OHTSURU, A., NAKAO, K., SHIMA, M., NAKATA, K., WATANABE, K., ISHII, N., KIMURA, N. & NAGATAKI, S. (1992). Expression in human hepatocellular carcinoma of nucleoside diphosphate kinase, a homologue of the nm23 gene product. *J. Natl Cancer Inst.*, **84**, 1349-1354.

NEUBAUER, A., NEUBAUER, B. & LIU, E. (1990). Polymerase chain reaction based assay to detect allelic loss in human DNA: loss of beta-interferon gene in chronic myelogenous leukemia. *Nucleic Acids Res.*, **18**, 993-998.

O'BRYAN, J.P., FRYE, R.A., COGSWELL, P.C., NEUBAUER, A., KITCH, B., PROKOP, C., ESPINOSA III, R., LE-BEAU, M.M., EARP, H.S. & LIU, E.T. (1991). axl, a transforming gene isolated from primary human myeloid leukemia cells, encodes a novel receptor tyrosine kinase. *Mol. Cell. Biol.*, **11**, 5016-5031.

POLYMEROPOULOS, M.H., XIAO, H., RATH, D.S. & MERRIL, C.R. (1991). Dinucleotide repeat polymorphism at the human gene of the light and heavy chains of myeloperoxidase glycoprotein (MPO). *Nucleic Acids Res.*, **19**, 1961.

POSTEL, E.H., BERBERICH, S.J., FLINT, S.J. & FERRONE, C.A. (1993). Human c-myc transcription Factor PuF identified a nm23-H2 nucleoside diphosphate kinase, a candidate suppressor of tumor metastasis. *Science*, **261**, 478-480.

RADINSKY, R., WEISBERG, H.Z., STAROSELSKY, A.N. & FIDLER, I.J. (1992). Expression level of the nm23 gene in clonal populations of metastatic murine and human neoplasms. *Cancer Res.*, **52**, 5808-5814.

ROCHLITZ, C.F., HEIDE, I., DEKANT, E., NEUBAUER, A., SCHMIDT, C.A., NEUHAUS, P., HUHN, H. & HERRMANN, R. (1993). Molecular alterations in a patient with Turcot's syndrome. *Br. J. Cancer*, **68**, 519-523.

ROSENGARD, A.M., KRUTZSCH, H.C., SHEARN, A., BIGGS, J.R., BARKER, E., MARGULIES, I.M., KING, C.R., LIOTTA, L.A. & STEEG, P.S. (1989). Reduced Nm23/Awd protein in tumour metastasis and aberrant Drosophila development. *Nature*, **342**, 177-180.

SAMBROOK, J., FRITSCH, E.F. & MANIATIS, T. (1989). *Molecular Cloning*. Cold Spring Harbor Laboratory Press: Cold Spring Harbor, NY.

STAHL, J.A., LEONE, A., ROSENGARD, A.M., PORTER, L., KING, C.R. & STEEG, P.S. (1991). Identification of a second human nm23 gene, nm23-H2. *Cancer Res.*, **51**, 445–449.

WANG, L., PATEL, U., GHOSH, L., CHEN, H.C. & BANERJEE, S. (1993). Mutation in the nm23 gene is associated with metastasis in colorectal cancer. *Cancer Res.*, **53**, 717–720.

YAMAGUCHI, A., URANO, T., FUSHIDA, S., FURUKAWA, K., NISHIMURA, G., YONEMURA, Y., MIYAZAKI, I., NAKAGAWARA, G. & SHIKU, H. (1993). Inverse association of nm23-H1 expression by colorectal cancer with liver metastases. *Br. J. Cancer*, **68**, 1020–1024.